# Assessment of training and mentoring for DR-TB care decentralization in Tanzania

**DOI:** 10.1186/s12960-021-00600-4

**Published:** 2021-04-26

**Authors:** Dennis Lyakurwa, Johnson Lyimo, Christiaan Mulder, Puck T. Pelzer, Inge Koppelaar, Marleen Heus

**Affiliations:** 1 KNCV Tuberculosis Foundation, Off-Haille Sellassie Road, Plot 8&10 Oysterbay, P.O.Box 11013, Dar es salaam, Tanzania; 2 Ministry of Health of Health, Community Development, Gender, Elderly and Children, P.O.Box 743, Dodoma, Tanzania; 3 KNCV Tuberculosis Foundation, The Hague, The Netherlands; 4 Amsterdam Institute for Global Health and Development, Amsterdam University Medical Center, Amsterdam, The Netherlands

**Keywords:** MDR-TB treatment, HCWs capacity building, Treatment outcomes

## Abstract

**Introduction:**

Drug-resistant TB (DR-TB) care shifted from centralized to decentralized care in Tanzania in 2015. This study explored whether DR-TB training and mentoring supported healthcare workers’ (HCWs) DR-TB care performance.

**Methods:**

This mixed study assessed HCWs’ DR-TB care knowledge, the training quality, and the mentoring around 454 HCWs who were trained across 55 DR-TB sites between January 2016 and December 2017. Pre- and post-training tests, end-of-training evaluation, supervisor’s interviews, DR-TB team self-assessment and team focus group discussion were conducted among trained HCWs. Interim and final treatment results of the national central site and the decentralized sites were compared.

**Results:**

HCW’s knowledge increased for 15–20% between pre-training and post-training. HCWs and supervisors perceived mentoring as most appropriate to further develop their DR-TB competencies. Culture negativity after 6 months of treatment was similar for the decentralized sites compared to the national central site, 81% vs 79%, respectively, whereas decentralized sites had less loss to follow-up (0% versus 3%) and fewer deaths (3% versus 12%). Delays in laboratory results, stigma, and HCWs shortage were reported the main challenges of decentralized care.

**Conclusions:**

Training and mentoring to provide DR-TB care at decentralized sites in Tanzania improved HCWs’ knowledge and skills in DR-TB care and supported observed good interim and final patient treatment outcomes despite health system challenges.

**Supplementary Information:**

The online version contains supplementary material available at 10.1186/s12960-021-00600-4.

## Background

TB remains a major cause of morbidity and mortality in Tanzania. The prevalence of multidrug resistant tuberculosis (MDR-TB) in Tanzania was estimated at 1.0% among new TB patients and 4.1% among retreatment patients in 2017 [1]. Only 6% and 10% of the estimated MDR-TB patients were enrolled on treatment for 2016 and 2017, respectively [2, 3] highlighting the large treatment gap for DR-TB patients.

In 2009, Tanzania started Programmatic Management of Drug Resistant TB (PMDT) using a centralized approach whereby all patients were managed at one national site; the Kibong’oto Infectious Diseases Hospital (KIDH). In 2015, the National Tuberculosis and Leprosy Program (NTLP) developed the *Implementation Framework for Expanded Decentralization of MDR-TB Services in Tanzania* [4] as a step to decentralize DR-TB diagnosis and care. This adopted an approach intended to provide DR-TB treatment on an ambulatory basis utilizing community-based providers so as to reduce hospital admissions for DR-TB patients. To implement the decentralization of DR-TB services, NTLP designed a competency-based training and mentoring package for the facility HCWs.

Studies in other high burden countries in Africa and Asia showed that ambulatory, community-based DR-TB care is more acceptable for patients and their family members and equally or more effective with high treatment success rates and less risk for defaulter than hospital-based care [5–9]. The importance of sufficient and qualified HCWs to provide quality (TB) care has been profoundly investigated [10–14]. However, the impact of training and supervision on HCWs performance is understudied, in addition it has been found difficult to measure due to the array of factors that influence HCWs’ performance [11, 12, 14, 15].

The objective of our study was to explore whether DR-TB training and mentoring improved HCWs performance in providing DR-TB care at DR-TB treatment initiation sites in Tanzania.

## Methods

### Study design

**Fig. 1:**
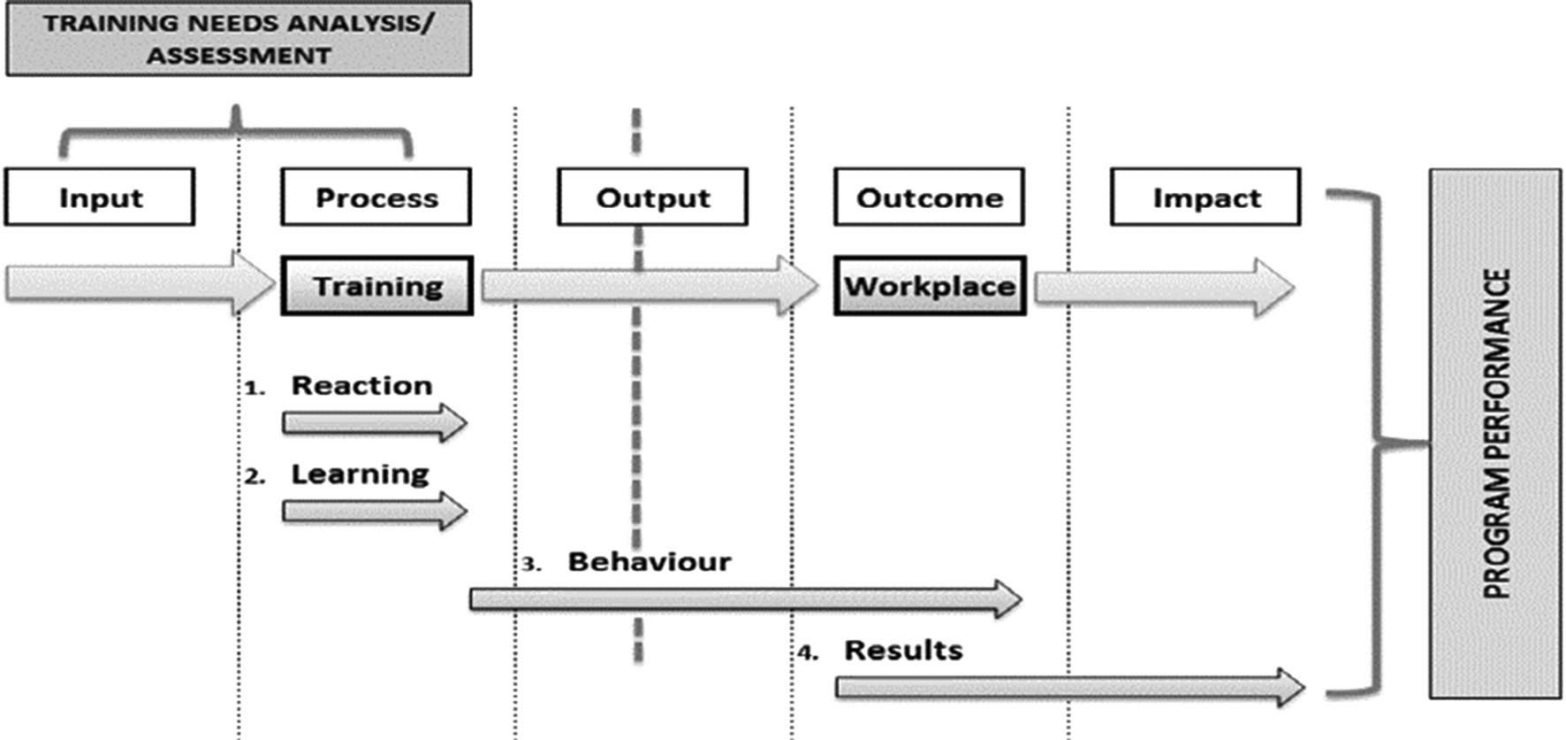
Kirkpatrick’s evaluation model

We conducted a mixed study design using Kirkpatrick’s evaluation model (Fig. 1) [16]. The quantitative component of the study consisted of: characteristics of DR-TB treatment initiation sites and trained HCWs, pre- and post-test scores, end-of-training evaluation results, and DR-TB treatment outcomes as a proxy for HCWs performance. The qualitative component consisted of: the DR-TB teams’ self-assessment, focus group discussions with the team, and the supervisor’s interview. The quantitative component was carried out first to assess the quality of training followed by qualitative interviews to elicit health workers perception, attitudes and practices on provision of decentralized DR-TB care.

The DR-TB treatment outcomes were assigned by a team of physicians working at Kibong’oto Infectious Diseases Hospital based on the patient’s progress (i.e., based on adherence to treatment and signs of clinical improvement), and mycobacterial culture results. The treatment outcomes were recorded as cured, treatment completed, died, treatment failure, lost to follow-up or not evaluated as adapted from WHO DR-TB definitions [17]. Favorable treatment outcome: was defined as a combination of treatment completed and cured and unfavorable treatment outcome means a combination of deaths, lost to follow, culture positive and not evaluated. Where available, culture was used classify the interim (culture conversion at month six of treatment) and final treatment outcomes at the end of treatment.. During the time of the study, the cultures were performed at the Central TB Reference Laboratory (CTRL) and at zonal TB laboratories using Lowenstein–Jensen (LJ) medium with a turnaround time of 3–8 weeks as per Tanzanian operational guidelines for management of drug resistance TB [18].

### Study setting

The interviews for HCWs were conducted at the one national central site (Kibong’oto Infectious Diseases Hospital) and 14 decentralized sites which were selected purposefully to include sites with high patient volumes, treating 286/327 (87%) of patients in 2016 and 2017 when the study was conducted. Treatment outcomes of 327 DR-TB patients were analyzed for 2016 and 2017 to explore how training supported the level of care in the decentralized sites.

### Study population

**Fig. 2:**
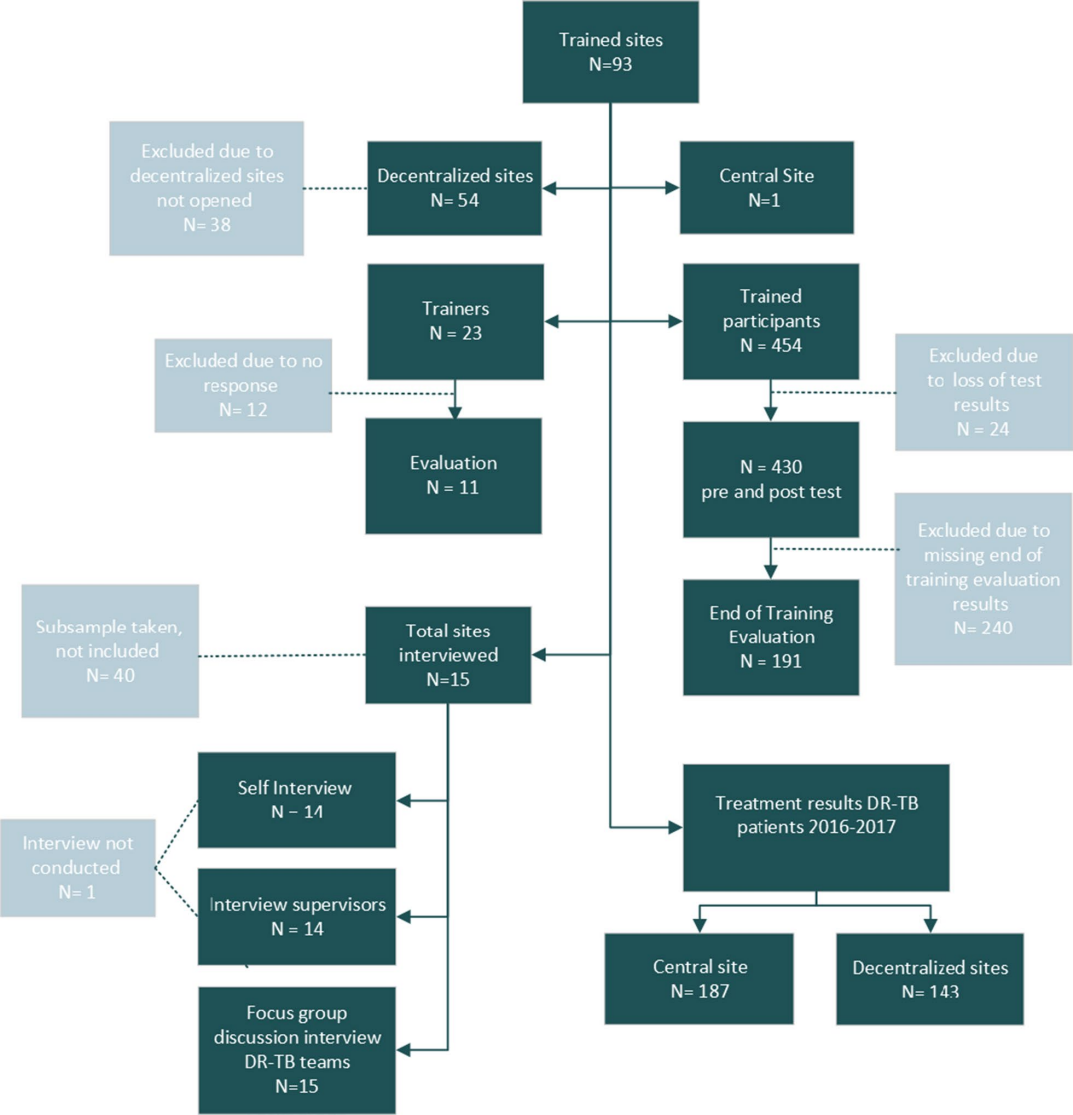
Flowchart inclusion and exclusion of decentralized DR-TB treatment sites that were trained and evaluated

This study included 454 HCWs from 93 DR-TB initiation sites that were trained between January 2016 and December 2017 by a team of 23 national trainers. Outcomes of patients enrolled in the year prior to training (2015) were used for comparison. The performance of 55 sites out of 93 sites was assessed as these were providing DR-TB care by December 2017. DR-TB teams from 15 sites were interviewed in a focus group discussion, 14 of which completed the self-assessment questionnaire and 14 had their supervisors interviewed to collect qualitative information about DR-TB teams’ performance on the job (Fig. 2). Interviews were conducted in 2018 and data analysis was done in 2019. This long duration after the training was to accommodate for outcome results of trained sites that take 24 months for the whole cohort to complete treatment after treatment initiation.

**Fig. 3:**
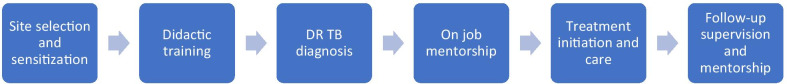
DR-TB care decentralization process in Tanzania

As part of the decentralization process, the NTLP selected the decentralized sites, assessed their readiness to start DR-TB care, supported improvement of the sites’ infrastructure and equipment, and built HCWs capacity to provide DR-TB diagnosis and care through a didactic training and on-job mentoring (Fig. 3).

The didactic training consisted of a 5-day comprehensive training using the newly developed “training modules for DR-TB initiation sites” (Additional file 1). The training methodology was competency based and interactive. We trained an interdisciplinary DR-TB team consisting of: a Regional TB and Leprosy Coordinator (RTLC), a District TB and Leprosy Coordinator (DTLC), a Clinician, a DOT nurse, a Laboratory Technician/Technologist, a Pharmacist, and a Social Welfare Officer.

On-job mentorship started once a DR-TB patient is diagnosed in their respective district, a mentoring team from KIDH, provided an additional 5-day mentoring on bedside clinical and nursing care and second-line drugs supply chain management.

Treatment of the DR-TB patients was done according to Operational Guidelines for Management of Drug-resistant TB in Tanzania, second Edition (2015) [19].

Quarterly supervision was provided by a team of four people from national, regional and district-level staff. During the supervision, the identified gaps were mentored on site. The next quarter supervision team also made a follow-up of previous visit action points before starting a new supervision.

### Data collection and analysis

For qualitative data collection, we used semi-structured interviews for DR-TB team self-assessment and supervisors and a tool for DR-TB team focus group discussions interview. Qualitative data were collected on HCWs performance and the relevance of the training and mentoring one year after the training was conducted to the 15 DR-TB sites. All available members of the DR-TB teams in 15 sites participated in the focus group discussion interviews at the DR-TB health facility. The interviewer (BS) was an independent researcher not involved in DR-TB care, training or mentoring. BS interviewed and facilitated focus group discussions, making use of standardized tools (Additional file 1). KNCV officer (DL) collected and analyzed the questionnaires filled by the trainers of trainers (Additional file 1). DR-TB team members of 14 sites self-assessed their performance and 14 of these DR-TB teams had their supervisor participate in the semi-structured interviews. Collected transcripts from semi-structured interviews were coded by two authors MH and DL. Coded inputs were then categorized to generate themes using thematic analysis.

**Fig. 4:**
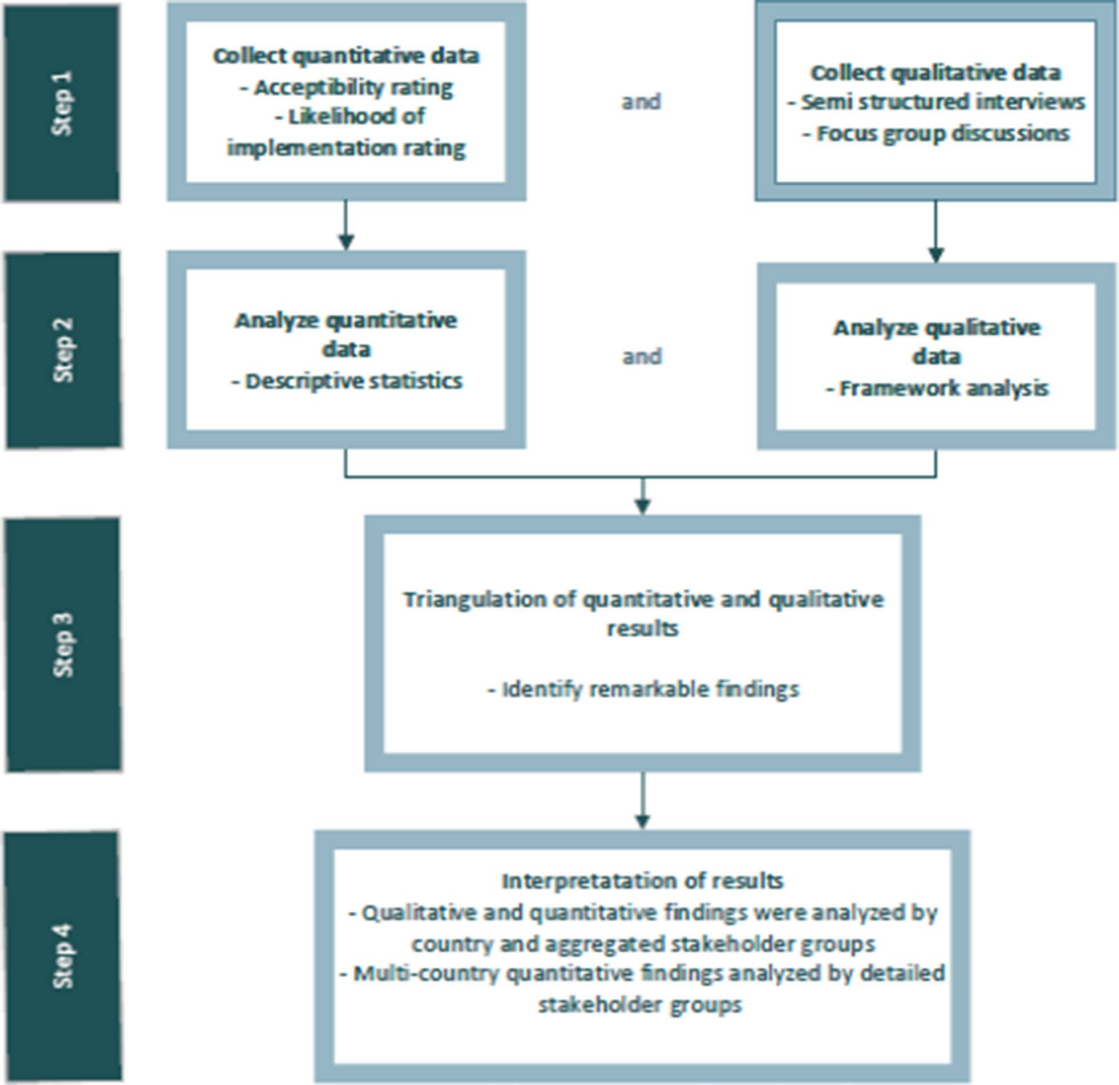
Flowchart for qualitative and quantitative data collection and analysis

For the quantitative component, characteristics of the DR-TB treatment initiation sites, enrolled DR-TB patients and trained HCWs were collected. Training impact was assessed using the Kirkpatrick’s evaluation model. Interim and final DR-TB treatment outcomes were compared between the central and decentralized sites and were extracted from the DR-TB enhanced cohort review system. Frequencies per demographic variables such as cadre and gender were presented for the different training groups. Training evaluation scores for method and content modules were measured on a Likert scale: 1 = poor, 2 = satisfactory, 3 = very good and 4 = excellent. The pre- and post-training test scores were compared before and after the training using comparison of means and a paired *t*-test. A *p*-value of < 0.05 was considered statistically significant. Multivariable logistic regression was used to determine if training was a predictor of outcomes. Data analysis was performed using Stata Software version 16.1 (StataCorp LP, College Station, TX, USA). We used a composite variable for the final outcome whereby favorable outcomes were a combination of cure and treatment completion rates and unfavorable outcomes comprised death, lost to follow-up, not evaluated and treatment failure (Fig. 4).

## Results

### Quantitative results

The number of DR-TB initiation sites increased from 1 national DR-TB site (KIDH) to 55 sites from January 2016 to December 2017. Decentralized DR-TB sites are distributed all over the country, with a concentration in the Eastern and Northern part of the country. Most sites are in urban areas where most patients are found (Additional file 1). In our study, we selected 15 sites to evaluate the effect of training and mentoring on HCWs performance (Additional file 1). These sites treated 286/328 (87%) DR-TB patients in 2016 and 2017.

A total of 449 MDR-TB patients were enrolled in treatment between 2015 and 2017. Decentralization begun in 2016 with 23% of patients in decentralized sites which increased to 62% in 2017. During the same period, 430 trained HCWs were analyzed for performance of the training and mentoring after excluding 24 HCWs who missed their results (Fig. 2, Table 2).

The distribution of the patient characteristics between patients treated in the central and decentralized sites were comparable for age, sex, treatment history, HIV status, and nutritional status, but not for patient residence as the patients treated at the central site were more often from a rural residence in 2017 (Table 1).

**Table 1:** Demographics of patients treated at decentralized and centralized sites in 2016 and 2017 compared to 2015

Year of enrollment (*N*)	2015; (*N* = 119)	2016; (*N* = 121)	2016; (*N* = 36)	2017; (*N* = 66)	2017 (*N* = 107)	Total (*N* = 449)
Type of initiating facility	KIDH (100%)	KIDH (77%)	Decentralized (23%)	KIDH (38%)	Decentralized (62%)	
Variable						
Age						
≤ 30 years	39 (33)	37 (31)	11 (30)	12 (18)	39 (36)	138 (31)
31–50 years	54 (46)	64 (53)	19 (53)	42 (64)	51 (48)	230 (51)
> 50 years	25 (21)	20 (17)	6 (17)	12 (18)	17 (16)	80 (18)
Sex						
Male	85 (71)	85 (70)	27 (75)	44 (67)	70 (65)	311 (69)
Female	34 (29)	36 (30)	9 (25)	22 (33)	37 (35)	138 (31)
Treatment history						
New	35 (30)	29 (24)	7 (20)	15 (23)	36 (34)	122 (27)
Retreatment	81 (70)	92 (76)	29 (80)	51 (77)	70 (66)	323 (72)
HIV status						
HIV co-infected	35 (29)	42 (35)	13 (36)	25 (39)	35 (33)	150 (33)
On ART	33 (94)	40 (95)	13 (100)	23 (92)	35 (100)	144 (32)
Patient residence						
Urban	79 (66)	70 (58)	29 (81)	19 (29)	76 (71)	273 (61)
Rural	40 (34)	51 (42)	7 (19)	47 (71)	31 (29)	176 (39)
Nutritional status						
< 18.5	57 (59)	73 (60)	25 (69)	32 (48)	42 (39)	229 (51)
> 18.5	40 (41)	48 (40)	11 (31)	34 (52)	65 (61)	198 (44)
HFs trained and initiated MDR-TB treatment						
Yes	1 (100)	1 (100)	14 (78)	1 (100)	35 (75)	55 (100)
No						
MDR-TB patients initiated with sites trained						

Out of these 430 trained HCWs, 226 (53%) were male, 132 (32%) were regional and district TB program coordinators, 33 (8%) TB direct observed treatment (DOT) nurses, 43 (10%) laboratory staff, 73 (17%) other clinicians, 78 (18%) other nurses, 39 (9%) pharmacists, and 28 (7%) social workers (Table 2).

**Table 2:** Characteristics of trained HCWs

Training year	2016, *N* (%)	2017, *N* (%)	Total
Total participants (*N*)	111 (26)	319 (74)	430
Cadre/role (%)			
TB Coordinator (RTLC, DTLC, TB/HIV Officer)	34 (31)	102 (32)	136 (32)
TB Nurse (DOT Nurse)	19 (17)	14 (4)	33 (8)
Lab Staff	13 (12)	30 (9)	43 (10)
Clinician	19 (17)	54 (17)	73 (17)
Nurse	5 (4)	73 (23)	78 (18)
Pharmacy Staff	12 (11)	27 (8)	39 (9)
Social Worker	9 (8)	19 (6)	28 (7)
Gender (%)			
Male	56 (50)	170 (53)	226 (53)
Female	55 (50)	149 (47)	204 (47)
Average score			
Pre-training test	42	41	41
Post-training test	60	64	62
*p*-value*	0.0001	0.0001	0.0001
Type of health facility (*N* = 55)			
Hospital	14 (70)	18 (51)	33 (58)
Health Center	2 (10)	11 (32)	13 (24)
Dispensary	4 (20)	6 (17)	10 (18)

*DOT* directly observed treatment, *NA* not available

A multidisciplinary team of 21 national trainers was formed: clinicians (10), nurses (7), and laboratory staff (4). Eleven out of 21 trainers, participated in this study and were; clinicians (6), laboratory staff (3) and nurses (2).

The pre-training/post-training test scores showed that trained HCWs had an average increase of 15–20 points during the course and this was statistically significant with *p* < 0.001 (Table 2).

**Fig. 5:**
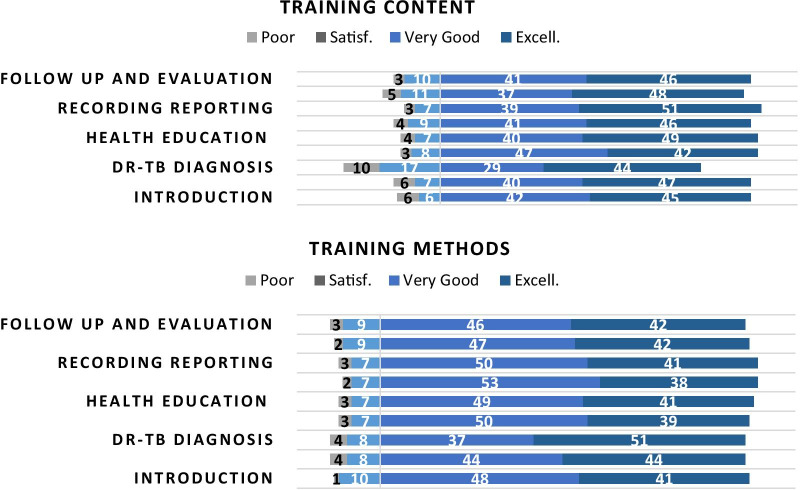
Training content and method evaluation scores by participants

Participants’ end-of-training evaluation results showed that most trained HCWs highly appreciated the course content and methodology (Fig. 5). The modules “Health Education”, “Supportive Supervision” and “Recording & Reporting” had highest scores, however the score differences among the modules were limited. The score differences among the training groups were not substantial.

449 DR-TB patients initiated treatment from 2015 to 2017. For interim results (after 2 months of treatment) decentralized sites had high proportion of culture conversion (72%) compared to centralized site (50%) in 2016, however there was no difference in the 2 month culture conversion between central and decentralized sites in 2017. After 6 months of treatment, culture conversion was comparable between decentralized and centralized sites in 2016 and 2017. For final outcomes, cure rates were higher for decentralized (86%) than KIDH (58–69%) in 2016 and 2017. Likewise, deaths and loss to follow-up were lower for decentralized sites compared to KIDH in the same years (Table 3).

**Table 3:** Interim and final outcomes at decentralized vs central (KIDH) sites; in 2016 and 2017 compared to 2015

Initiating facility	Centralized—KIDH			Decentralized	
Year	2015	2016	2017	2016	2017
Interim outcomes after 2 months					
Culture conversion 2 months, *n* (%)	60 (55)	54 (50)	34 (52)	26 (72)	51 (50)
Interim outcomes after 6 months					
Culture negative, *n* (%)	101 (92)	105 (96)	58 (92)	34 (94)	94 (91)
Loss to follow-up, *n* (%)	3 (3)	1 (1)	2 (3)	0 (0)	0 (0)
Died, *n* (%)	12 (10)	15 (12)	8 (12)	1 (3)	3 (3)
Interim outcomes after 12 months					
Loss to follow-up, n (%)	5 (4)	6 (5)	3 (5)	0 (0)	2 (2)
Died, n (%)	6 (5)	5 (4)	5 (8)	2 (6)	2 (2)
Final treatment results					
Treatment success rate, *n* (%)	88 (74)	94 (77)	47 (71)	32 (89)	96 (90)
Cured, *n* (%)	71 (60)	84 (69)	38 (58)	31 (86)	92 (86)
Treatment complete, *n* (%)	17 (14)	10 (8)	9 (14)	1 (3)	4 (4)
Died, *n* (%)	22 (18)	21 (17)	14 (21)	4 (11)	7 (7)
Lost to follow-up, *n* (%)	7 (6)	5 (4)	3 (5)	0 (0)	2 (1)
Treatment failure, *n* (%)	1 (1)	1 (1)	1 (2)	0 (0)	2 (2)
Not evaluated, *n* (%)	1 (1)	0 (0)	1 (2)	0 (0)	0 (0)

Comparing predictors of unfavorable outcomes in decentralized vs centralized site in 2016 and 2017 (Table 4), we found BMI (< 18.5 kg/m^2^) was a significant predictor of unfavorable outcomes among patients at the national central site [adjusted OR–aOR; 3.1, *p* = 0.005] similarly to HIV-coinfection among decentralized treated patients (aOR; 4.2, *p* = 0.02). Older ages [31–50 years (aOR 2.2, *p* = 0.02) & > 50 year (aOR 2.6, *p* = 0.02) and centralized treatment delivery (aOR 2.9 *p* = 0.006) were associated with unfavorable outcomes.

**Table 4:** Predictors of favorable outcomes (deaths, LTFU, Rx failure, and NE) between decentralized vs centralized sites in 2016 and 2017

Initiating facility	Centralized—KIDH (*N* = 306)				Decentralized sites (*N* = 143)				Combined (*N* = 449)			
Variable	Crude OR	*p*-value	Adjusted OR	*p*-value	Crude OR	*p*-value	Adjusted OR	*p*-value	Crude OR	*p* value	aOR (95% CI)	*p* value
			(95% CI)		(95% CI)		(95% CI)					
Treatment cohort												
2016	1				1				1		1	
2017	1.4	0.32	1.81	0.14	0.91	0.88	0.78	0.74	0.77	0.078	1.6	0.2
	(0.7–2.7)		(0.8–3.9)		(0.22–3.1)		(0.18–3.3)		0.58–1.02		0.75–3.4	
Sex												
Female	1				1				1		1	
Male	2.1	0.057	2.14	0.073	0.94	0.91	0.79	0.73	1.3	0.28	1.2	0.38
	(0.9–4.9)		(0.92 -5)		(0.3–2.9)		(0.2–3.1)		0.79–2.2		0.7–2.3	
HIV												
Positive	1.2	0.52	1.07	0.87	4.7	0.007	4.2	0.02	1.6	0.03	1.5	0.12
	(0.6–2.5)		(0.48–2.3)		(1.5–14.7)		(1.2–15)		1.04–2.6		0.8–2.6	
Negative	1				1				1		1	
BMI												
≥ 18.5	1				1				1		1	
< 18.5	3.2	0.002	3.1	0.005	0.99	0.988	0.97	0.97	2.3	0.002	2.2	0.004
	(1.6–6.8)		(1.3- 7)		(0.3–2.89)		(0.28- 3.3)		1.3–4		1.3–3.8	
Residence												
Urban	1.2	0.47	1.63	0.23	1.5	0.54	2.1	0.175	0.99	0.98	1.3	0.3
	(0.6–2.4)		(0.6–4.)		(0.4–5.6)		(0.39–11)		0.6–1.5		0.7–2.6	
Rural	1				1					1	1	
Patient group												
New	1				1				1		1	
Retreatment (FLD)	1.14	0.74	0.98	0.99	0.61	0.39	0.48	0.23	1.05	0.85	0.8	0.5
	(0.5–2.1)		(0.4–2.43)		(0.2–1.8)		(0.14–1.6)		0.6–1.7		0.4–1.4	
Staff training												
Yes	N/A				1							
No					1.25	0.78	1.29	0.79	0.5	0.42	1.01	0.9
					(0.25–6.1)		(0.19–8.4)		0.12–2.4		0.19–5.8	
Age categories												
< 30 years	1				1				1		1	
31–50 years	3.09	0.02	3.05	0.03	4	0.08	3	0.2	1.9	0.02	2.2	0.02
	(1.2–8)		(1.1–8.4)		(0.8–19)		(0.55–16)		1.1–3.5		1.1–4.1	
> 50 years	2.3	0.145	3.1	0.08	3.6	0.178	4	0.18	2.3	0.016	2.6	0.02
	(0.7–7.7)		(0.88–8.4)		(0.55–23)		(0.5–33)		1.1–4.7		1.1–5.9	
Treatment delivery												
Centralized	N/A				N/A				2.8	0.001	2.9	0.006
Decentralized	1								1.5–5.1		1.3–6.2	

Covariates controlled for in the adjusted analysis are treatment cohort, BMI, HIV, patient group, age categories, treatment delivery, residence, sex and staff trained

### Qualitative results

The trained HCWs of the 15 DR-TB sites qualified the training course as comprehensive, relevant, good, practical and helpful and that it did not focus on clinical topics only. The course gave them confidence and skills to provide treatment to DR-TB patients. Five DR-TB teams mentioned that the “New Drugs and Regimens” session was confusing. Teams suggested regular updates on (the frequent) changes in DR-TB care and train new staff members which are needed due to frequent staff rotation. (Additional file 1).

All 11 trainers evaluated the quality of the training as good: as it included all aspects of decentralized DR-TB care and perceived that trained HCWs have learned from this course. Eight out of 11 trainers assumed that the trained HCWs were ready to initiate DR-TB treatment. Three out of 11 trainers expressed their hesitations mainly due to lack of adequate facility equipment and infrastructure and insufficient Infection Prevention and Control practice. Some trainers observed that trained HCWs lacked confidence in the quality of care at their facility and fear DR-TB because they have no experience in treating DR-TB patients and assume having a high risk to be infected by them (Additional file 1).

Supervisors assessed DR-TB teams’ performance after the training as very good (5 supervisors) to good (10 supervisors) concluding that trained HCWs had developed their DR-TB knowledge during the training (Additional file 1).

Nine of the 15 DR-TB sites received mentoring after DR-TB training, of which four teams felt they were inadequately mentored, and two teams perceived the mentoring more as control than support of the DR-TB team. During mentoring with facility multidisciplinary teams, the interviewer noted the frequency and duration of the mentoring varied from team to team between a ½ h visit to more than 5 days on the job training and, from *when a new patient comes* to four times a year. There was no standardized mentoring approach and the mentoring was strongly focused on the clinicians and DOT nurses. Mentoring and supportive supervision were perceived the most appropriate way for most DR-TB teams to develop further their competencies. DR-TB teams mentioned *They showed how to initiate treatment and brought common understanding among the team* and *We saw how the mentor performed in handling patients and medication; he built our confidence*.

The four trainers that had also a mentoring role, mentioned that mentoring has reinforced what the trained HCWs learned in the training because their workplaces afforded them with more comfortable learning environment, was more hands-on and focused on observed HCWs gaps.

The DR-TB teams self-evaluated their performance between fair and excellent on the different aspects of quality performance (Additional file 1). There were no substantial differences among the DR-TB teams. Teams were most satisfied with their performance on (1) confidence to provide DR-TB care; (2) ensure availability and quality of DR-TB drugs; (3) give health education and (4) provide patients support. Teams had concerns about their performance on (1) timely and accurate laboratory test results; (2) timely and quality clinical care; (3) quality recording and reporting and (4) quality and regular supportive supervision. The teams proposed to train more HCWs, include facility managers in the training and provide ongoing mentorship. They suggested to update HCWs regularly on new developments, increase punctuality to provide laboratory results and involve the whole laboratory team in this. They proposed monthly DR-TB meetings, training and mentoring to ensure that all HCWs are competent and up to date.

DR-TB teams’ supervisors frequently mentioned the DR-TB teams’ strengths in treatment initiation, providing the correct drugs and drugs dosage, patient management and care, laboratory investigations and patient follow-up. The main challenges that DR-TB teams faced according to their supervisors are mainly in the field of stigma, facility infrastructure, drug delivery, delays in culture results, HCWs shortage and HCWs capacity building (Table 5). Supervisors suggested to further build DR-TB team capacity in patient data entry, management of side effects and short regimens. DR-TB teams need to develop their competencies further by practice to gain confidence. *The team doesn’t get many patients and needs more practice in initiating treatment*, said one of the supervisors. Regular and quality supervision, reminding HCWs of what they have learned in the course, is a must for professional development. Continuous medical education for HCWs that were not trained yet and sharing of documents were also brought forward.

**Table 5:** The main challenges that trained DR-TB teams face in providing DR-TB care as mentioned by supervisors (*N* = 14) and trained DR-TB teams (*N* = 15)

Challenge	DR-TB team challenges mentioned by supervisors—citation	Challenges mentioned by the DR-TB team—citation
Stigma in the community and stigma among DR-TB patients	*There is still stigma towards patients who wear masks*	*HCWs: Stigma is very high irrespective of the Health Education every Wednesday {1 participant}* *We are told by patients to go without mask due to fear of being stigmatized. (1 participant}*
Inadequate infrastructure	*There are inadequate waiting areas, inadequate space for patient management and poor ventilation*	
Inadequacy in the system of drug delivery	*There is shortage and delays in (short regimen) drugs*	
Delays in laboratory results	*Sometimes there are no culture results at all* *It can take 5 – 6 months* to receive the results	*Delays in getting investigation results especially culture results (even in KIDH) {9 participants}* *Modules for GeneXpert need replacement {1 participant}*
Working conditions and staff capacity building are not up to standard	*2 staff were transferred out and no new staff brought-in* *Few staff but involved in other programs without replacement* *No extra-duty to staff working on holidays* *in KIDH 32 more staff members were employed, their challenge is to build the capacity of this new staff* *There is a lack of refresher training*	*Modalities for home visits are not clear {2 participants}*

## Discussion

This mixed study in Tanzania suggested that a “national DR-TB continuous learning approach” including a standardized training package, on-job mentoring, and follow-up supervision can support good interim and final treatment results at decentralized DR-TB sites, compared to the already existing central site. Mentoring and supervision by the national central site were considered an essential and integral component of HCWs decentralized capacity building which likely supported the favorable performance of decentralized sites that were not trained as these were mentored by the central team before initiating MDR-TB treatment. Studies from Kyrgyzstan and Nigeria (10), Bangladesh (21), South Africa (9) and Indonesia (14) confirm that quality training, mentoring and supervision of frontline workers are essential to provide quality (DR-) TB care.

Our study findings are in line with those from Kenya, South Africa and Ethiopia which also adopted TB ambulatory care models [20–22]. In addition, an Ugandan study reported that DR-TB decentralization was preferred and acceptable to patients, families and communities [7].

HCWs DR-TB knowledge to initiate, monitor, and provide treatment to patients increased between pre-training and post-training implying the added value of the DR-TB training package. After the training and mentoring, HCWs acknowledged that they had skills to initiate DR-TB treatment and provide quality care with good treatment outcomes. These results were discussed and endorsed in a meeting with the PMDT technical working group. Studies in other countries also showed that the treatment outcomes of decentralized care are comparable to centralized care, but the risk for defaulting is lower at the decentralized sites because of the patient centered approach of the mode of treatment [6, 8, 9, 23].

Decentralization was undertaken in a phased manner and it took time for health facilities to get ready (after implementing a readiness assessment checklist and/or renovations to TB clinic/wards), updating the national and regional supply chain system (N95 respirators and medicines), HCWs training, on-job mentoring and supervision. Trained HCWs, trainers and supervisors mentioned that the DR-TB training was important to prepare HCWs for the decentralized DR-TB care. The practical and interactive training methods, allowing HCWs to practice and ask questions, have facilitated trained HCWs’ learning [24]. Other studies [10–12, 24] confirmed that training is an important intervention to build HCWs capacity especially when HCWs is faced with new tasks and responsibilities like the HCWs in the DR-TB decentralized sites.

To ensure up-to-date quality trainings, National TB Programs need to evaluate their training courses systematically on HCWs’ knowledge, job performance and program outcome, and frequently update their training packages based on these evaluation results, new guidelines and practices. Evaluation of HCWs performance and patient outcomes at the facility level often does not take place because of lack of priority, funding, experience, and tools [10, 15, 25]. NTPs also need to invest in training of trainers to build a pool of trainers that are competent to work optimally with the training packages [10, 14, 26].

All stakeholders acknowledged that mentoring and supervision were important to reinforce HCWs’ performance after the training. In comparison to training, mentoring and supervision have the additional value of focusing on skills building, addressing HCWs’ gaps, and repeating the key information shared in the training, and therefore reinforcing learning [12, 14, 24]. Mentoring and supervision were not standardized and differed in duration, frequency, and approach. Therefore, the effect of mentoring and supervision on HCWS’ capacity building may have been different among the health facilities.

Tanzania has chosen for a strongly decentralized model of DR-TB care, to provide care close to patients’ homes. However, at the beginning, the number of DR-TB patients was limited leading to DR-TB initiation centers with few patients only. There was a fear that HCWs in these sites would not build their DR-TB experience and risk losing the knowledge and skills they had been trained in. Monitoring the quality of DR-TB care in these small-scale DR-TB initiation centers needs extra attention and on-job HCWs mentoring is crucial. It might even be more effective to choose for on-job mentorship approach first (when a site has a patient) followed later by a comprehensive training. A dialogue is needed among the main stakeholders in DR-TB care, on the appropriate level of DR-TB care decentralization and investments needed for HCWs development and infrastructure.

Trained DR-TB HCWs at the decentralized sites mentioned several bottlenecks that hindered their performance. To provide quality decentralized DR-TB care, these bottlenecks need to be addressed. Training, mentoring and supervision alone are not enough and need to be part and parcel of health systems strengthening interventions, to be effective [7, 10].

A limitation of this study was that it was not designed to compare treatment outcomes between sites with and without DR-TB training, for which a cluster randomized trial would have been the preferred approach. This would, however, raise ethical concerns considering the necessity of highly trained HCWs to provide quality DR-TB care. As such this study does not provide a causative link between a HCWs training program and HCWs behavior nor treatment outcomes, but it does support the merit of providing a standardized training package combined with on the job mentoring. Another limitation, due to budget constraints, was that patients were not interviewed to know how they perceive the quality of the DR-TB care to explain the variability of their treatment outcomes. In addition, the qualitative data were collected 1 year after the training which might have introduced recall and information bias. Only 11 out of 23 trainers assessed the quality of the training and the training package which could bias the results. Four trainers were supervisors as well which could have biased the results of the self-assessment.

The mixed study findings displayed the quality of DR-TB training necessary to build the, HCWs’ knowledge and skills to support the HCW’s performance and the DR-TB treatment results. The qualitative data have given additional insights in HCWs and their supervisors’ perceptions on the quality of DR-TB care at decentralized sites that would not have been possible using a quantitative study only.

In conclusion, the success of decentralization in Tanzania is the outcome of investment in programmatic management in DR-TB, the enhanced cohort review process and capacity building. Therefore, we recommend NTPs to invest consistently in HCWs capacity building enabling HCWs to provide quality DR-TB care and implement innovations in DR-TB diagnosis and treatment. The focus should be on the day-to-day mentoring, which will contribute to flexible and continuous capacity building of HCWs in DR-TB care. The recently developed DR-TB Quality Improvement Tool (Additional file 1) could support NTPs, supervisors, and mentors in achieving this.

## Supplementary Information

**Additional file 1.** Supplement file.

## Data Availability

The data used and or analyzed during this research are stored online using Microsoft teams. The data can be accessed through a permission from the corresponding authors.
