# Supplementary material for: Assessment of training and mentoring for DR-TB care decentralization in Tanzania
Source: Hum Resour Health. 2021 Apr 26;19:56. doi: 10.1186/s12960-021-00600-4 (PMC8077954; doi:10.1186/s12960-021-00600-4)
Supplement: Supplementary file 1 — Additional file 1. Supplement file. [file 12960_2021_600_MOESM1_ESM.docx]

**Additional file 1**

*Modules and sessions of the DR-TB training for teams at DR-TB initiation sites*

|  | **Module** | **Session** |
| --- | --- | --- |
| 1 | Introduction | 1.Introduction and pre-training test |
| 2 | Programmatic Management of Drug Resistant TB | 2.1 Concepts of DR-TB  2.2 DR-TB Epidemiology  2.3 The organizational structure of DR-TB services in Tanzania |
| 3 | Diagnosis of DR-TB | 3.1 Identification of presumptive DR-TB  3.2 Laboratory tests and interpretation of test results |
| 4 | Management of DR-TB patients | 4.1 Evaluation of the patient  4.2 DR-TB Treatment regimens  4.3 DR-TB Patient monitoring  4.4 Infection Prevention and control  4.5 DR-TB Patient support  4.6 DR-TB Medical rounds  4.7 Co-morbidities and DR-TB in special situations |
| 5. | Health education, adherence counselling and treatment care plan | 5.1 Basics of DR-TB patients’ education  5.2 Adherence and Treatment Care Plan |
| 6 | Supportive supervision and mentoring | 6.1 Procedures and tools for DR-TB supervision  6.2 DR-TB mentoring at ambulatory sites |
| 7 | Recording and Reporting | 7.1 Recording and reporting tools and procedures  7.2 Practicing recording and reporting of DR-TB data |
| 8 | Logistics Management | 8.1 Supply chain management  8.2 Pharmacy practice |
| 9 | Follow up and evaluation | 9.1 Action planning  9.2 Post- training test and course evaluation |

*Tools to collect and document qualitative information*

**Semi structured interviews on training and mentoring**

Target group: DR-TB teams at initiation sites

Tool: semi structured interview for focused group discussion

Interviewer: independent researcher

| *Name of DR-TB Treatment initiation site:* | |  |
| --- | --- | --- |
|  | Month + year of staff DR-TB training |  |
|  | Number of staff trained in DR-TB diagnosis and treatment |  |
|  | When (year and month) did they do this DR-TB training? |  |
|  | What are the functions of staff trained (clinician, nurse, lab technician etc.)? |  |
|  | Was staff trained before or after opening of the DR-TB site? |  |
|  | Reasons for training staff after opening the DR-TB site |  |
|  | Reasons for not training staff at all |  |
|  | If not trained:  How were staff prepared on their new tasks/ responsibilities? |  |
|  | How do staff evaluate the training? |  |
|  | What were staff’s priority learning results? |  |
|  | How important is this training to start DR-TB care? |  |
|  | If not important: why not? |  |
|  | Were staff mentored after the training? Frequency and how? |  |
|  | If mentored: for how long and by whom? |  |
|  | If not mentored: why not? |  |
|  | If mentored:  How did they perceive the mentoring?  Did they benefit from it?  What were the benefits? |  |
|  | If they didn’t benefit from the mentoring: what had they expected from the mentoring? |  |
|  | DR-TB team from Initiation Site: When does the DR-TB team refer patients to the DR-TB Treatment Site? Is this in line with guidelines/recommendations? |  |

*Self-assessment on team performance by the DR-TB* *teams*

Target group: DR-TB teams

Tool: self-assessment scoring list

Facilitated by independent researcher

|  | **Performance indicator** | **Performance Scale:**  1 – Poor, 2 – Fair,  3 – Good,4 – Excellent | **Performance improvements suggested by the team** |
| --- | --- | --- | --- |
|  |  |  |  |
| 0 | **Confident to provide DR-TB care**  How confident is the team in providing DR-TB care? |  |  |
| 1 | **Timely laboratory tests**  For DR-TB initiation sites: Time for diagnosis < 24 hours (Smear& GeneXpert) and 8 – 12 weeks (culture)  For DR-TB treatment sites: Timely laboratory tests |  |  |
| 2 | **Accurate laboratory tests provided** |  |  |
| 3 | **Timely clinical care**:  For DR-TB initiation sites: Time from diagnosis to treatment <24 hours  For DR-TB treatment sites: Clinical care immediate at arrival of the patient |  |  |
| 4 | **Quality clinical care**  Appropriate diagnosis  Appropriate physical examination  Appropriate dosing  Assess and address appropriately drug side effects and co-morbidities.  Treatment care plan implemented and updated |  |  |
| 4 | **Quality DR-TB drugs**:  Available  Stored appropriately  Drug forms and reports filled in correctly |  |  |
| 5 | **Infection Prevention and Control in place**  IPC plan available  IPC plan implemented and monitored |  |  |
| 6 | **Continuous health education for patients and DOT provider** |  |  |
| 7 | **Patient support discussed and organized** |  |  |
| 8 | **Quality recording and reporting**  Timely  Complete  Accurate recording and reporting |  |  |
| 9 | **Continuous and supportive supervision is provided**:  By the DR-TB team of the DR-TB Initiation site to the DR-TB treatment site  By the DR-TB team of the Treatment Site to the DR-TB treatment supporters |  |  |
| 10 | **DR-TB team is well informed and the activities are well coordinated** |  |  |

*Semi structured Interview on DR-TB team’s performance*

Target group: Supervisors of DR-TB teams at DR-TB initiation sites

Tool: semi structured interview

Interviewer: independent researcher

**Name of the DTLC:**

**Name of the DR-TB Treatment Initiation Site:**

|  | **Name of trainer** | Comment |
| --- | --- | --- |
|  | How does the DR-TB team perform? |  |
|  | What are the strengths of the DR-TB team? |  |
|  | What must the team improve? |  |
|  | Was the DR-TB team confident and competent sufficiently prepared after the training, to start DR-TB care in their facility? If not: what else did/ do, they need? |  |
|  | When does the DR-TB team of the DR-TB Initiation Site refer patients to the DR-TB Treatment Site? Is this in line with guidelines/recommendations? |  |
|  | Did the team implement the action plan they developed in the DR-TB training? What were they not able to implement and why? |  |
|  | How do you further develop the DR-TB team’s performance? |  |

*Questionnaire on training and mentoring*

Target group: trainers

Tool: Questionnaire sent out by mail

Information collected by: KNCV officer

|  | **Name of trainer** | Comment |
| --- | --- | --- |
|  | How many DR-TB Initiation Sites trainings did you facilitate? |  |
|  | Which session(s) did you facilitate? |  |
|  | Did you completely follow the newly developed training package? Why and why not?  If not: where did you deviate from the training protocol and why? |  |
|  | How do you perceive the quality of the DR-TB Initiation Sites training package? **Poor or Fair or Good**  Please tell why. |  |
|  | How do you perceive the quality of the DR-TB Initiation sites trainings you participated in? **Poor or Fair or Good**  Please tell why. |  |
|  | Were, according to you, the participants sufficiently prepared to start DR-TB care in their facility, after this training?  If yes: explain what make you think so.  If no: explain what make you think so. |  |
|  | Were you involved in mentoring / supervision of the DR-TB teams? |  |
|  | Did the mentoring reinforce the topics that the team learned in the training? Give some examples. |  |

*Spatial overview of DR-TB sites in Tanzania by December 2017*

The bullets represent the Geographic Information System (GIS) coordinates of the DR-TB sites and the number of sites.


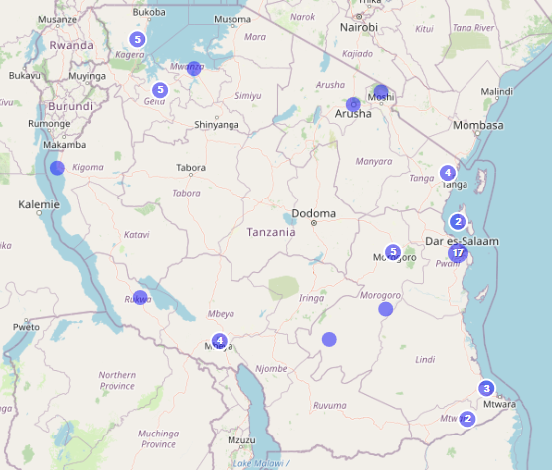


*Overview of staff trained per cadre, and staff not trained in the 15 sites interviewed*

Of the 15 sites included in the qualitative study 8 started providing treatment after the training, the remaining 7 sites had already started providing treatment before staff did the official training. Out of the 237 DR-TB staff in these 15 sites, 86 (36%) staff were trained, 11 (13%) were pharmacists, 23 (27%) clinicians, 32 (37%) DOT nurses, 16 (19%) lab staff, 11 (13%) pharmacists and 4 (5%) social workers.

|  | **Site** | **Number of Cadre trained** | | | | | **Total Number staff trained** | **Number of staff not trained** |
| --- | --- | --- | --- | --- | --- | --- | --- | --- |
|  |  | **Clinician** | **DOT Nurse** | **Laboratory technicians** | **Pharmacists** | **Social Worker** |  |  |
| 1 | Mbagara Rangi Tatu | 3 | 3 | 2 | 1 | 1 | 10 | 6 |
|  |  |  |  |  |  |  |  |  |
| 2 | Magereza Ukonga Dispensary | 2 | 4 | 0 | 0 | 0 | 6 | 7 |
|  |  |  |  |  |  |  |  |  |
| 3 | Sinza Hospital | 1 | 4 | 5 | 1 | 2 | 13 | 3 |
|  |  |  |  |  |  |  |  |  |
| 4 | Tambuka Reli Dispensary | 1 | 2 | 1 | 1 | 0 | 5 | 10 |
|  |  |  |  |  |  |  |  |  |
| 5 | Amana District Hospital | 1 | 3 | 1 | 0 | 0 | 5 | 9 |
|  |  |  |  |  |  |  |  |  |
| 6 | Bagamoyo District hospital | 1 | 1 | 0 | 1 | 0 | 3 | 17 |
|  |  |  |  |  |  |  |  |  |
| 7 | Bukoba Regional Referral Hospital | 2 | 1 | 0 | 1 | 0 | 4 | 7 |
| 8 | Sekou Toure Hospital | 2 | 3 | 4 | 1 | 0 | 10 | 1 |
|  |  |  |  |  |  |  |  |  |
| 9 | Bukombe District hospital | 1 | 1 | 0 | 1 | 0 | 3 | 23 |
| 10 | Chato District hospital | 1 | 1 | 0 | 0 | 0 | 2 | 30 |
| 11 | Katoro Health center | 1 | 1 | 0 | 0 | 0 | 2 | 12 |
|  |  |  |  |  |  |  |  |  |
| 12 | KIDH | 3 | 5 | 1 | 3 | 0 | 12 | 9 |
| 13 | Ngamiani Health Center | 1 | 1 | 0 | 0 | 0 | 2 | 6 |
| 14 | Ruanda HC | 2 | 1 | 1 | 1 | 1 | 6 | 11 |
| 15 | Mbeya regional reference hospital | 1 | 1 | 1 | 0 | 0 | 3 | 29 |
| TOTAL | | 23 | 32 | 16 | 11 | 4 | 86 | 151 |

^KIDH: Kibong’oto Infectious Disease Hospital | DOT nurse : Direct Observed Treatment nurse^

*Overview of frequently mentioned reasons of the relevance of the DR-TB training among N=14 DR-TB teams*

|  | **Themes mentioned** | **Number of teams that mentioned this theme** | **Citation** |
| --- | --- | --- | --- |
| 1 | Skills to initiate and manage MDR-TB patients, including patient management | 9 | 1. *No patients died* 2. *No patients stopped treatment* 3. *Most patients adhere to treatment* 4. *We have good treatment outcomes* |
| 2 | Comprehensive course including all aspects of TB care | 4 |  |
| 3 | No longer fear for MDR-TB | 4 | 1. *“We had not seen an MDR-TB patient before* “ *and therefore were afraid of these patients and their disease* 2. *At first, we were running away from those patients* 3. *We have no longer fear for MDR-TB patients* |
| 4 | Staff confident to treat MDR-TB patients at decentral sites | 3 | *After the training we were more confident to diagnose and treat MDR-TB patients* |

*Overview of trainers’ assessment on the quality and relevance of the DR-TB training and the training package, N=11*

*X= Number of times mentioned*

| **Strengths of the DR-TB training** | **Strengths of the DR-TB training package** | **Participants are ready to start DR-TB treatment because** |
| --- | --- | --- |
| 1. Includes all aspects of decentralized DR-TB care (4x) 2. Most of the sites managed to initiate treatment (1x) 3. Participants have learnt from this course: post-training scores were higher than pre-training AND the training content was well understood (1x) 4. Work in Site-teams worked out very well, teams could discuss real life problems and solutions (1x) 5. Methodology was good (1x) 6. Training was better organized than in previous years (1x) | 1. Facilitators’ and Participants’ Manual are well organized and good quality (2x) 2. Training package is comprehensive and includes relevant information (6x) 3. Different teaching methods were used (2x) | 1. Training is comprehensive with both theory and practical sessions, and visit to KIDH (1x) 2. Participants participated actively in the course and learnt (1x) 3. Support through supervision and mentorship will build further their skills and confidence (3x) 4. Most sites had already patients on continuation phase. They are confident to initiate treatment (1x) 5. Some participants had already experience in TB control (1x) |
|  | **Suggested improvements in the training package** | **Participants not completely ready to start DR-TB treatment because** |
|  | 1. Minor content updates in specific modules (3x) 2. Minor improvements of slides in specific modules (3x) 3. Align some slides better with the Facilitator’s manual (1x) 4. Improve hard copy printing (1x) | 1. Lack of or inadequate equipment /infrastructure (4x) 2. Insufficient IPC practice (4x) 3. Lack of confidence in the care at their facility (2x) 4. Fear DR-TB (2x) 5. Insufficient psychological preparation of HCWs (1x) |

*Overview of supervisors’ evaluation of staff performance after the training.*

Supervisors assessed DR-TB teams’ performance after the training as very good (5x) to good (10x) concluding that trained HCWs had developed their DR-TB knowledge during the training

| **Staff performance after the training** | **Number of supervisors that mentioned this** | **Citation** |
| --- | --- | --- |
| The team is competent and confident to provide DR-TB care | 12 | 1. *They are very confident now* 2. *Training helped them; they can initiate treatment now* 3. *During mentoring KNCV created awareness among all staff about DR-TB* 4. *They are not afraid of DR-TB patients anymore, as they were before the training* 5. *They gained confidence almost 90%* 6. *They are competent and confident to fill in well the registers, to initiate treatment and to mentor other sites*. |
| Not fully competent yet to provide DR-TB care | 3 | 1. *They need an update on Short Treatment Regimens* 2. *Competent on long treatment not competent on STR yet* |
| Difficult to evaluate | 1 | *I am their supervisor for only 2 months* |

*Self-assessment DR-TB team performance (*N= 15) teams

|  | **Performance indicator** | **Performance score , assessed by teams** | | | | **Performance improvements suggested by the team** |
| --- | --- | --- | --- | --- | --- | --- |
|  |  | 1 | 2 | 3 | 4 |  |
| 0 | **Confident to provide DR-TB care** |  |  | 8 | 7 | More training for some HCWs |
| 1 | **Timely laboratory tests** |  | 6 | 7 | 2 | More punctual to provide facility lab results |
| 2 | **Accurate laboratory tests provided** |  | 1 | 10 | 4 | Some baseline investigation need more time, so all baseline investigations to be available at one point |
| 3 | **Timely clinical care** |  | 4 | 8 | 3 | Ongoing training and mentorship |
| 4 | **Quality clinical care** |  | 2 | 10 | 3 | Ongoing training, mentorship |
| 4 | **Quality DR-TB drugs** |  |  | 6 | 9 | Mentorship/training for newly staffs at the site like Pharmacists. |
| 5 | **Infection Prevention and Control in place** |  | 2 | 10 | 3 | To strengthen  IPC plan implementation and monitoring |
| 6 | **Continuous health education for patients and DOT provider,** |  |  | 9 | 6 | Keep it up |
| 7 | **Patient support discussed and organized** |  |  | 10 | 5 |  |
| 8 | **Quality recording and reporting** |  | 2 | 10 | 1 | Team work is highly needed with tools familiarization to all team members through mentorship |
| 9 | **Continuous and supportive supervision is provided**: |  | 1 | 9 | 5 | DR-TB  Team needs regular training, when the changes or regimen has been changed |
| 10 | **DR-TB team is well informed and the activities are well coordinated** |  | 1 | 11 | 3 | Ongoing refresher training and mentorship |

Performance scale: 1 – Poor, 2 – Fair, 3 – Good, 4 – Excellent

*Table 12:* DR-TB Patients’ Clinical Management

***Quality Improvement Tool***

# Acknowledgments

This “Quality Improvement Tool” (QI Tool) was developed as an output of the “Core Bedaquiline Coordination Project” for Challenge TB Project (CTB) under the APA4 workplan.

The project team adapted the earlier tools developed by the European Respiratory Society, European Centre for Disease Control, and CTB Indonesia on the clinical management of tuberculosis (TB) patients. This QI Tool was upgraded for use by a new target group, namely: supervisors and monitoring specialists working at the National TB programs and partner organizations.

This QI Tool was pilot-tested in countries in Central Asia Region, Eastern Europe, South East Asia and Africa, at the district and facility levels. Health professionals (from NTPs, KNCV, PATH) in the countries, where the QI Tool was pilot-tested, made a huge contribution to improve the initial draft versions of the tool. Their enthusiastic participation during the pilot tests gave the QI Tool the benefit of their ideas and experience and helped to keep it focused on the practical needs and challenges of health providers who manage patients with drug-resistant TB (DR-TB).

The Global Health Bureau, Ofﬁce of Health, Infectious Disease and Nutrition (HIDN), USAID, ﬁnancially supports this guide through Challenge TB under the terms of Agreement No. AID-OAA-A-14-00029. This guidance document is made possible by the generous support of the American people through the USAID. The contents are the responsibility of Challenge TB, and do not necessarily reﬂect the views of USAID or the United States Government.


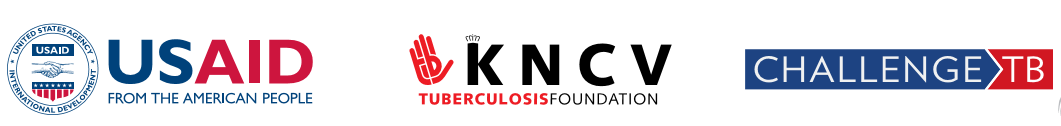


# Introduction to the Quality Improvement Tool

The objective of this Quality Improvement (QI) Tool is to assess patients’ diagnostic and treatment pathways in order to identify potential bottlenecks leading to an optimization of the daily clinical practice for all DR-TB patients being managed at the respective health facility.

This QI Tool is to be used to document patients’ clinical management, to facilitate on-the-job training of staff, and informing discussions on quality improvement of the daily clinical practice.

The tool consists of three parts:

***PART*** ***1*** collects general information on the respective health facility and indicates what diagnostic and monitoring services are available at the respective health facility.

***PART 2*** collects information from files and patients’ treatment cards/charts to give an insight into the day-to-day clinical management of the patients (from diagnosis to completion of treatment) in order to initiate a discussion with staff leading to agreed next steps and timelines to improve the quality of patient clinical management at the respective health facility. Each worksheet can collect data about five patients. If you have time and need to assess more than five patients, additional worksheets developed for PART 2 accordingly can be used.

***PART 3*** provides recommendations for on the job training and improvements.

The QI Tool is designed as a participatory job aid- "learning through doing”, e.g. systemic analysis of the current situation and identified issues, discussions, problem solving, and planning and taking actions. Users learn through interaction with each other and conducting immediate on-the-spot discussions amongst

themselves in small groups. It is designed to move from learning to action(s) for improving the quality of the clinical management of the DR-TB patients at the respective health facility.

*Quality Improvement Tool*

# PART 1: HEALTH FACILITY

| [DD/MM/YEAR] of the **PREVIOUS** | NAME/ POSITION Auditor | PHONE NUMBER/EMAIL Auditor |
| --- | --- | --- |
| [DD/MM/YEAR] of the **CURRENT** | NAME/ POSITION Auditor | PHONE NUMBER/EMAIL Auditor |

| FACILITY | FACILITY ADDRESS/ PHONE NUMBER |  |
| --- | --- | --- |
| NAME/ POSITION contact person of the facility | PHONE NUMBER/EMAIL contact person of the facility |  |

| - 1. ***FACILITY CHARACTERISTICS***   Pls tick [**x** or **√**] to indicate what type of a health facility is assessed | | | |
| --- | --- | --- | --- |
| Facility | ☐ general with TB OPD | ☐ X MDR-TB facility |  |
| Type of services | ☐ out-patient | ☐ in- patient |  |
| For which age group services are provided | ☐ adults | ☐ children |  |

| - 1. ***AVAILABILITY OF TB DIAGNOSTIC LABORATORY SERVICES***   Pls tick [**x** or **√**] to indicate what type of tests are available | |
| --- | --- |
| **Smear microscopy**  Yes, at the facility ☐ Not available  ☐ Yes, transferred (transported) to another facility | **Xpert MTB/RIF**  Yes, at the facility ☐ xNot available  ☐ Yes, transferred (transported) to another facility |
| Comments:  *Describe the transportation system*  *Mnazi facility vehicles* | Comments:  *Describe the transportation system*  **Mnazi facility vehicles** |
| **1^st^-line LPA**  ☐ Yes, at the facility ☐ Not available  ☐ Yes, transferred (transported) to another facility | **2^nd^- line LPA**  ☐ Yes, at the facility ☐ Not available  ☐ Yes, transferred (transported) to another facility |
| Comments:  *Describe the transportation system*  Sample transportation by motor cyclist | Comments:  *Describe the transportation system*  Idem |
| **Culture on solid media (L-J)**  ☐ Yes, at the facility ☐ Not available  ☐ Yes, transferred (transported) to another facility | **Culture on liquid media (MGIT)**  ☐ Yes, at the facility ☐ Not available  ☐ Yes, transferred (transported) to another facility |
| Comments:  *Describe the transportation system*  idem | Comments:  *Describe the transportation system*  idem |
| **1^st^-line pDST MGIT**  ☐ Yes, at the facility ☐ Not available  ☐ Yes, transferred (transported) to another facility | **2^nd^-line pDST MGIT**  ☐ Yes, at the facility ☐ Not available  ☐ Yes, transferred (transported) to another facility |
| Comments:  *Describe the transportation system*  idem | Comments:  *Describe the transportation system*  **i**dem |
| **1st-line pDST L-J**  ☐ Yes, at the facility ☐ Not available  ☐ Yes, transferred (transported) to another facility | **2^nd^- line pDST L-J**  ☐ Yes, at the facility ☐ Not available  ☐ Yes, transferred (transported) to another facility |
| Comments:  *Describe the transportation system*  idem | Comments:  *Describe the transportation system*  idem |
| **Other:** ……………………………….  ☐ Yes, at the facility ☐ Yes, transferred (transported) to another facility ☐ Not available | |
| Comments:  *Describe the transportation system* | |

| - 1. ***AVAILABILITY OF MONITORING TESTS***   Pls tick [**x** or **√ if YES**] to indicate what tests are available | | | | |
| --- | --- | --- | --- | --- |
| Tests | Yes, at the facility | Yes, transferred (transported) to another facility | Not available | Comments |
| Full blood count |  |  |  |  |
| Liver enzymes |  |  |  |  |
| Serum creatinine |  |  |  |  |
| Potassium |  |  |  |  |
| Magnesium/ Calcium |  |  |  |  |
| Uric acid |  |  |  |  |
| Thyroid stimulating hormone |  |  |  |  |
| Blood glucose |  |  |  |  |
| Serum albumin |  |  |  |  |
| Lipase/amylase Lactic acid |  |  |  |  |
| HIV test |  |  |  |  |
| Viral load |  |  |  |  |
| CD4 |  |  |  |  |
| Hepatitis virus panel |  |  |  |  |
| Pregnancy test |  |  |  |  |
| Audiometry |  |  |  |  |
| ECG/ QTc calculation |  |  |  |  |
| Visual acuity |  |  |  |  |
| Color vision |  |  |  |  |
| X-Ray |  |  |  |  |
| *Comments:*  *If partially available, e.g. no FBC but rapid test for Hb only, put in comments* | | | | |

| - 1. ***INFECTION CONTROL MEASURES IMPLEMENTED AT THE FACILITY***   Pls tick [**x** or **√**] to indicate measures indicated | | | |
| --- | --- | --- | --- |
| Are DS-TB patients separated from DR-TB patients?  **In- patient**  ☐Yes ☐ N/A  **Out-patient**  ☐No ☐ N/A | Are RR-/ MDR-TB separated from pre-XDR-TB/XDR-TB patients?  **In- patient**  ☐Yes ☐ N/A  **Out-patient**  ☐Yes ☐ N/A | Are there isolation rooms available for respiratory infection patients (in the ward)?  ☐Yes ☐No | Are respiratory infectious patients supplied with surgical masks?  **In- patient**  ☐ Yes ☐ N/A  **Out-patient**  ☐ Yes ☐No |
| *Comments:* Transportation of patients is done *in a pick car to manage control infection* | | | |

| - 1. ***DRUG STOCKS AT THE HEALTH FACILITY***   Pls tick [**x** or **√**] to indicate measures indicated | |
| --- | --- |
| Has there been a stock out drugs used for DR-TB treatment during last 3 months?  ☐Yes ☐ No | Has there been a stock out of Bdq, Dlm, and repurposed drugs (Mfx, Lfx, Cfz, Lzd) during last 3 months?  ☐Yes ☐ No |
| *Comments:* | |

# Part 2: Patients

***Pls. fill in the UNIT or DEPARTMENT of the FACILITY, where information is being collected:***

***Introduction***

From the DR-TB patients currently on treatment in this facility, randomly select 5-10 patients, and ask the assisting facility staff to pick out patients’ treatment cards files. The number of patients will depend on the amount of time you have to review their information, but the minimum should be five. Enter the patient’s registration no. in the in the table below, so that the answers and conclusions in the subsequent sections can be traced back to the individual patient.  It is possible that the information requested may also be in the DR-TB Register.

To randomly select patients, use the function RANDBETWEEN in Excel. For instance, if there are 100 patients in the *Masterlist* and they are numbered 1 to 100, type: “=RANDBETWEEN(1,100)”. It will give you a random number between 1 and 100. You can repeat the RANDBETWEEN function 10 times and take the numbers that are indicated. If the numbers are 6-36, type “=RANDBETWEEN(6,36)”. If you copy the content of the cell to another cell, it will give you another random number.

**Please note that the RANDBETWEEN return value changes every time you change anything in the Excel file. Should you want to store the randomly returned numbers, then please copy them to Word. If you want to copy them to Excel, then make sure that you choose the Paste Special > Past Values option.*

**Example**

Suppose that there are 100 patients and you want to review 10 patient files, then, on average, 1 in 10 patients should be selected. You can either repeat the RANDBETWEEN function 10 times and take the numbers that are returned (in case of duplicate numbers you will need to repeat), or (simpler), ask for a random number between 1 and 10 and add 10 to it several times. So, if the number 3 is returned, then the 3^rd^, 13^th^, 23^rd^, etc. patients’ files should be reviewed.

| ***Patients***  For each selected patient (1-5), pls. write down the ID or registration number | | | | | | |
| --- | --- | --- | --- | --- | --- | --- |
|  | Patient 1 (P1) | Patient 2 (P2) | Patient 3 (P3) | Patient 4 (P4) | Patient 5 (P5) | Notes |
| Registration number |  |  |  |  |  |  |
| Gender (M/F) |  |  |  |  |  |  |
| Age |  |  |  |  |  |  |

| ***2.1. DIAGNOSIS AND TURN-AROUND TIME OF RESULTS AT TREATMENT INITIATION***  ***Please compare results jotted down in patients’ cards/charts with source documents, e.g., laboratory test results, etc.***  To fill in the data below, pls. **DATE**, **0=Unknown** in case the information is missing in the patients’ file **or N/A= not applicable** not applicable for any reason. | | | | | | | |
| --- | --- | --- | --- | --- | --- | --- | --- |
|  | | **P1** | **P2** | **P3** | **P4** | **P5** | Comments |
| Date registered as presumptive DR-TB or date of 1^st^ consultation (last episode) |  |  |  |  |  |  |  |
| Date Xpert MTB/RIF sample collected (last episode) | |  |  |  |  |  |  |
| Date Xpert MTB/RIF results | reported by laboratory |  |  |  |  |  |  |
|  | received by clinician |  |  |  |  |  |  |
| Turn-around-time (T-A-T) (days) Xpert MTB/RIF* | |  |  |  |  |  |  |
| Date of referral from peripheral to treatment initiating center |  |  |  |  |  |  |  |
| Date of the treatment initiation |  |  |  |  |  |  |  |
| Date of baseline SL LPA sample collected |  |  |  |  |  |  |  |
| Date of baseline SL LPA results | reported by laboratory |  |  |  |  |  |  |
|  | received by clinician |  |  |  |  |  |  |
| Turn-around-time (T-A-T) SL LPA* |  |  |  |  |  |  |  |
| Date of baseline culture sample collected | MGIT |  |  |  |  |  |  |
|  | LJ |  |  |  |  |  |  |
| Date of baseline culture result reported by laboratory | MGIT |  |  |  |  |  |  |
|  | LJ |  |  |  |  |  |  |
| Date of baseline culture result received by clinician | MGIT |  |  |  |  |  |  |
|  | LJ |  |  |  |  |  |  |
| Turn-around-time (T-A-T) baseline culture* | MGIT |  |  |  |  |  |  |
|  | LJ | 35 days |  |  |  |  |  |
| Date of baseline sample collected for 1^st^ line DST**)** | MGIT |  |  |  |  |  |  |
|  | L-J |  |  |  |  |  |  |
| Date of baseline 1^st^ line DST results reported by laboratory | MGIT |  |  |  |  |  |  |
|  | L-J |  |  |  |  |  |  |
| Date of baseline 1^st^ line DST results received by clinician | MGIT |  |  |  |  |  |  |
|  | L-J |  |  |  |  |  |  |
| T-A-T baseline 1^st^ line DST***** | MGIT |  |  |  |  |  |  |
|  | L-J |  |  |  |  |  |  |
| Date of baseline sample collected for 2^nd^ line DST | MGIT |  |  |  |  |  |  |
|  | L-J |  |  |  |  |  |  |
| Date of baseline 2^nd^ line DST results reported by laboratory | MGIT |  |  |  |  |  |  |
|  | L-J |  |  |  |  |  |  |
| Date of baseline 2^nd^ line DST results received by clinician | MGIT |  |  |  |  |  |  |
|  | L-J |  |  |  |  |  |  |
| T-A-T baseline 2^nd^ line DST ***** | MGIT |  |  |  |  |  |  |
|  | L-J |  |  |  |  |  |  |
| **Date of DR-TB treatment initiation** | |  |  |  |  |  |  |
| Time from presumptive DR-TB or Xpert MTB/RIF result to initiation of DR-TB treatment ** | |  |  |  |  |  |  |
| Comments:  P1: | | | | | | | |
| P2: | | | | | | | |
| P3: | | | | | | | |
| P4: | | | | | | | |
| P5: | | | | | | | |
| **TAT can be calculated by number of days between sample collection date and reported result.*  ***Number of days between “registered as presumptive DR-TB or date confirmed RR-TB by XpertMTB/RIF and date start of treatment”* | | | | | | | |

| ***2.2. TREATMENT PRESCRIPTION AND ADMINISTRATION***  To fill in the data below, please indicate **Yes, No, 0** (no data in the patients’ clinical records) **or N/A= not applicable** in case not applicable for any reason (e.g. not needed or not applicable at this moment). | | | | | | |
| --- | --- | --- | --- | --- | --- | --- |
|  | **P1** | **P2** | **P3** | **P4** | **P5** | Comments |
| Correct regimen choice (triage to STR or ITR) |  |  |  |  |  |  |
| Correct regimen design. STR Standard. ITR contains at least 5 (intensive phase) TB drugs deemed active |  |  |  |  |  |  |
| Right administration of drug as per label (dosage, frequency), according to the country guidelines |  |  |  |  |  |  |
| Adequate intensive phase  STR: intensive phase 4-6 months  ITR injectable (if included in the regimen) = 8 months |  |  |  |  |  |  |
| Adequate continuation phase*  **STR:** 5 months  **ITR:** minimum 12 months |  |  |  |  |  |  |
| Treatment adjusted according to the DST results or intolerance of drugs |  |  |  |  |  |  |
| Comments:  *Pls. indicate in the comments if patient’s regimen is without injectable, and borders of phases are difficult to distinguish*  P1: | | | | | | |
| P2: | | | | | | |
| P3: | | | | | | |
| P4: | | | | | | |
| P5: | | | | | | |
| *Duration of the treatment should be according to the national guidelines and sputum results (check the completion date). | | | | | | |

| ***2.3. TREATMENT RESPONSE MONITORING (BASELINE AND FOLLOW-UP TESTS)***  To fill in the data below, please indicate **Yes, No, ?** (if data are incomplete), **0** (no data in the patients’ clinical records), **or N/A= not applicable** in case not applicable for any reason (e.g. not needed or not applicable at this moment).  B= baseline, F= follow-up | | | | | | | | | | | |
| --- | --- | --- | --- | --- | --- | --- | --- | --- | --- | --- | --- |
|  | **P1** | | **P2** | | **P3** | | **P4** | | **P5** | | Comments |
|  | B | F | B | F | B | F | B | F | B | F |  |
| Weight |  |  |  |  |  |  |  |  |  |  |  |
| Body Mass Index |  |  |  |  |  |  |  |  |  |  |  |
| Smear |  |  |  |  |  |  |  |  |  |  |  |
| Culture |  |  |  |  |  |  |  |  |  |  |  |
| Repeated SL LPA/pDST |  |  |  |  |  |  |  |  |  |  |  |
| Chest X-ray |  |  |  |  |  |  |  |  |  |  |  |
| Comments:  P1: | | | | | | | | | | | |
| P2: | | | | | | | | | | | |
| P3: | | | | | | | | | | | |
| P4: | | | | | | | | | | | |
| P5: | | | | | | | | | | | |

| ***2.4. TREATMENT SAFETY MONITORING (BASELINE AND FOLLOW-UP TESTS)*** | | | | | | | | | | | |
| --- | --- | --- | --- | --- | --- | --- | --- | --- | --- | --- | --- |
| To fill in the data below, please indicate **Yes, No, ?** (if data are incomplete)**, 0** (no data in the patients’ clinical records) **or N/A= not applicable** in case not applicable for any reason (e.g. not needed or not applicable at this moment). | | | | | | | | | | | |
| B= baseline, F= follow-up | | | | | | | | | | | |
|  | **P1** | | **P2** | | **P3** | | **P4** | | **P5** | | Comments |
|  |  | F | B | F | B | F | B | F | B | F |  |
| Full blood count (FBC) |  |  |  |  |  |  |  |  |  |  |  |
| Liver enzymes |  |  |  |  |  |  |  |  |  |  |  |
| Serum creatinine |  |  |  |  |  |  |  |  |  |  |  |
| Potassium |  |  |  |  |  |  |  |  |  |  |  |
| Magnesium, Calcium |  |  |  |  |  |  |  |  |  |  | If potassium is abnormal |
| Uric acid |  |  |  |  |  |  |  |  |  |  |  |
| Thyroid stimulating Hormon (TSH) |  |  |  |  |  |  |  |  |  |  |  |
| Blood glucose |  |  |  |  |  |  |  |  |  |  |  |
| Serum Albumin |  |  |  |  |  |  |  |  |  |  |  |
| HIV test* |  |  |  |  |  |  |  |  |  |  | Pls indicate [+] or [-] |
| Hepatitis B |  |  |  |  |  |  |  |  |  |  | According to the country protocol |
| Hepatitis C |  |  |  |  |  |  |  |  |  |  | According to the country protocol |
| Pregnancy test |  |  |  |  |  |  |  |  |  |  |  |
| Audiometry |  |  |  |  |  |  |  |  |  |  |  |
| ECG / QTcF |  |  |  |  |  |  |  |  |  |  |  |
| Visual acuity |  |  |  |  |  |  |  |  |  |  |  |
| Color vision test |  |  |  |  |  |  |  |  |  |  |  |
| Other tests* |  |  |  |  |  |  |  |  |  |  |  |
| Pls indicate other tests (only if patient has specific clinical condition or specific treatment choices (e.g. patient is on Lzd or other options) | | | | | | | | | | | |
| Comments: | | | | | | | | | | | |
| P1: | | | | | | | | | | | |
| P2: | | | | | | | | | | | |
| P3: | | | | | | | | | | | |
| P4: | | | | | | | | | | | |
| P5: | | | | | | | | | | | |

| ***2.5. ADVERSE EVENTS***  To fill in the data below, please indicate **Yes, No, ?** (if data are incomplete)**, 0 (no data in the patients’ clinical records) or N/A= not applicable** in case not applicable for any reason (e.g. not needed or not applicable at this moment). | | | | | | |
| --- | --- | --- | --- | --- | --- | --- |
|  | **P1** | **P2** | **P3** | **P4** | **P5** | Comments |
| Serious adverse events detected |  |  |  |  |  |  |
| Serious adverse events managed properly |  |  |  |  |  |  |
| Ancillary drugs are available for serious adverse |  |  |  |  |  |  |
| Severe adverse events reported accordingly * |  |  |  |  |  | According to the country protocol: facility🡪 to national level🡪 to global level |
| Comments:  P1: | | | | | | |
| P2: | | | | | | |
| P3: | | | | | | |
| P4: | | | | | | |
| P5: | | | | | | |
| *** In case of a SAE a copy of the report should always be kept in the patients’ file. | | | | | | |

| ***Please fill section 2.6 for TB/HIV co-infected patients only, if they are HIV+, see section 2.4 otherwise indicate as N/A.***  ***2.6. MANAGEMENT TB/HIV CO-INFECTION ****  To fill in the data below, please indicate **Yes, No, ?** (if data are incomplete), **0 (no data in the patients’ clinical records) or N/A= not applicable** in case not applicable for any reason (e.g. not needed or not applicable at this moment).  B= baseline, F= follow-up | | | | | | |
| --- | --- | --- | --- | --- | --- | --- |
|  | **P1** | **P2** | **P3** | **P4** | **P5** | Comments |
| Viral load |  |  |  |  |  |  |
| CD4 count |  |  |  |  |  |  |
| Cotrimoxazole preventive therapy given |  |  |  |  |  |  |
| Correct timing of ART start |  |  |  |  |  |  |
| Correct ART regimen/ BDQ compatible |  |  |  |  |  |  |
| Correct Management of virological failure, timely switch ART |  |  |  |  |  |  |
| Comments:  P1: | | | | | | |
| P2: | | | | | | |
| P3: | | | | | | |
| P4: | | | | | | |
| P5: | | | | | | |

| ***2.7. PATIENT SUPPORT***  To fill in the data below, please indicate **Yes, No, ?** (if data are incomplete), **0 (no data in the patients’ clinical records) or N/A= not applicable** in case not applicable for any reason (e.g. not needed or not applicable at this moment). | | | | | | |
| --- | --- | --- | --- | --- | --- | --- |
|  | **P1** | **P2** | **P3** | **P4** | **P5** | Comments |
| Counselling and education on DR-TB done |  |  |  |  |  |  |
| Psychological assessment done |  |  |  |  |  |  |
| Social support assessment done |  |  |  |  |  |  |
| The individual support plan is developed, followed-up and updated |  |  |  |  |  |  |
| Comments:  P1: | | | | | | |
| P2: | | | | | | |
| P3: | | | | | | |
| P4: | | | | | | |
| P5: | | | | | | |

# PART 3: RECOMMENDATIONS FOR ON THE JOB TRAINING AND IMPROVEMENTS

| **Number** | **Recommendation** | **Responsible** | **Date**  **[dd/mm/yy]** | **Implemented**  **Yes/ No** |
| --- | --- | --- | --- | --- |
|  | Improvise isolation for MDRTB patients and procure ECG and audiometer for TB/MDR-TB monitoring. |  |  |  |
|  | Track the LPA, culture and DST results actively. |  |  |  |
|  | Continuous mentoring and follow up of specific findings will improve the performance |  |  |  |
|  | Use job aids (weight charts,app for calculating QTCf,creatinine clearance severity grading and schedules) which were mentored during this mentor |  |  |  |
|  |  |  |  |  |

*Consent for publication*
